# The Role of Autophagy in the Development of Pathological Conditions of the Body

**DOI:** 10.32607/actanaturae.23838

**Published:** 2023

**Authors:** U. S. Kench, S. S. Sologova, V. S. Prassolov, P. V. Spirin

**Affiliations:** Engelhardt Institute of Molecular Biology, Russian Academy of Sciences, Moscow, 119991 Russian Federation; Department of Pharmacology, Nelyubin Institute of Pharmacy, I.M. Sechenov First Moscow State Medical University (Sechenov University), Moscow, 119991 Russian Federation

**Keywords:** autophagy, apoptosis, cell death, lysosomes

## Abstract

Autophagy is the process of lysosomal elimination of the cell organelles, cytoplasmic sites, and pathogenic microorganisms that enter the cell. This process is associated with both cell death regulation and an increase in cell survival chances. Autophagy is involved in the development of various diseases (Crohn disease, cancer, atherosclerosis, etc.). For these reasons, it is of significant interest to establish the molecular targets involved in autophagy regulation and the factors that mediate its participation in pathogenesis. The review describes the potential molecular mechanisms involved in the regulation of autophagy, its contribution to the vital cell activity in a healthy organism, and pathologies.

## INTRODUCTION

Autophagy is the mechanism of removal of non-required and damaged organelles and cell cytosol regions. It is considered a compensatory response that is a result of the lack of nutrients in a cell, as well as a response to stress. In some cases, activation of autophagy leads to cell death. Thus, autophagy, on the one hand, protects cells from unfavorable external and internal factors, and, on the other hand, leads to cell death if it is impossible to save the cell and in case of viral or bacterial infection.

## 1. MECHANISMS OF AUTOPHAGY REGULATION

**Fig. 1:**
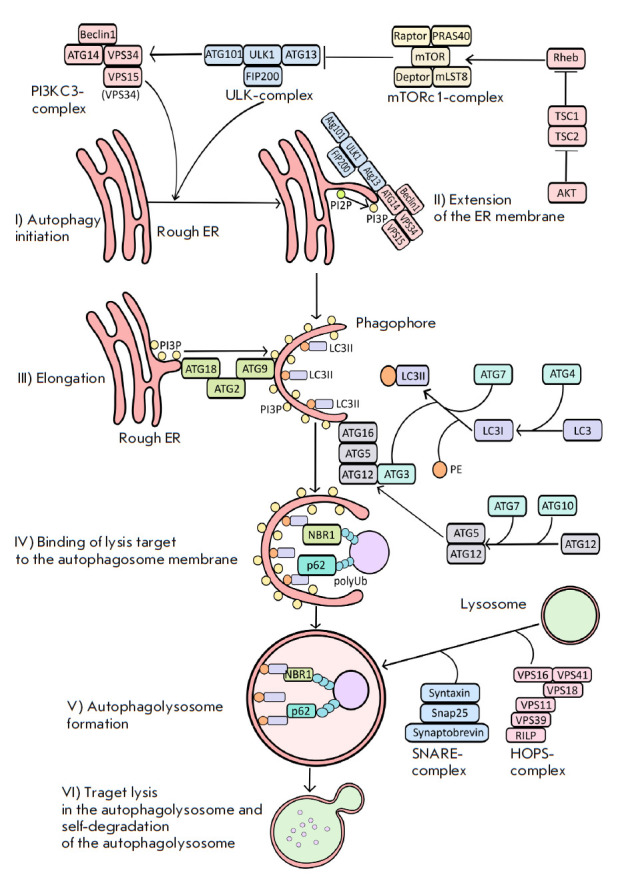
Schematic representation of autophagy. Autophagy is initiated by the inhibition of the mTORc1 complex, which prevents the ULK complex assembly (mTORc1 phosphorylates Atg13, which inhibits the assembly of the active ULK complex). Stage I – autophagy initiation. Stage II – endoplasmic reticulum membrane extension. Stage III – elongation. Stage IV – recruitment of the degradation target to the autophagosome. Stages V–VI – autophagolysosome formation and target lysis

During autophagy, an autophagosome is formed around the target to be degraded and the target then undergoes lysis. The following autophagy stages are usually distinguished: initiation, elongation, autophagosome formation, and formation of an autophagolysosome, followed by its degradation (*Fig. 1*).

**Stage I. **The initiation of autophagy begins with the extension of a section of the rough endoplasmic reticulum (ER) membrane, followed by its detachment. During initiation, the ULK complex is recruited to the outer ER membrane, leading to a change in the membrane structure. The ULK complex, which consists of the ULK1, Atg13, FIP200, and Atg101 proteins, is formed through dephosphorylation of the Atg13 and ULK1 proteins and simultaneous drop in the kinase activity of the mTORc1 complex. Dephosphorylation of Atg13 and ULK1 triggers the assembly of an active ULK complex [1]. Dephosphorylated Atg13, a ULK complex member, binds to Atg14, a member of the PI3KC3 complex (Vps34), while ULK1 phosphorylates the proteins Beclin1 (Atg6) and Vps34 and, thus, activates them (*Fig. 1*).

Beclin1 is the key protein for the PI3KC3 complex formation, while Vps34 is involved in the production of phosphoinositol triphosphate from the phosphoinositol diphosphate (PI2P) on the ER membrane surface. PI3P is required for the recruitment of the other proteins involved in phagophore formation and its subsequent transition to an autophagosome.

**Stage II. **The PI3KC3 complex (phosphatidylinositol- 3-kinase class 3), together with the ULK complex, promotes the extension of the ER membrane fragment and its subsequent detachment with the formation of a phagophore [1].

**Stage III. **Phagophore elongation includes modifications of its structure (enrichment of the phagophore membrane with PI3P, recruitment of LC3II), which are required for the binding of the target to be degraded to the autophagosome membrane. At this stage, the major conjugate complex Atg12/Atg5/Atg16 plays a key role. Conjugate formation begins with the processing of the ubiquitin-like protein Atg12, which is carried out by the ubiquitin-E1-like activating enzyme Atg7 [2] and the ubiquitin-E2-like enzyme Atg10 [3]. Atg5 and Atg16 then join the activated Atg12 (*Fig. 1*). The conjugate is also necessary to recruit the other proteins involved in elongation and ensure phagophore membrane extension [1, 2, 3].

**Stage IV. **At this stage, the target to be degraded is bound and positioned inside the autophagosome with the use of LC3II. LC3II is produced as a result of proteolytic cleavage of LC3 by the cysteine protease Atg4 with formation of the intermediate product LC3I. With the involvement of Atg7 and Atg3, LC3I interacts with phosphatidylethanolamine (PE) to form LC3II and anchor it on the phagophore membrane [2, 4, 5]. The Atg8 and GABARAP proteins possess functions that are similar to those of LC3II [5].

Simultaneously with LC3II processing, additional enrichment of the PI3P phagophore is carried out by the Atg9/Atg2/Atg18 conjugate, which transfers PI3P from the ER to the phagophore (*Fig. 1*, stage III) [6]. Atg13 initiates formation of the Atg9/Atg2/Atg18 conjugate.

For further autophagosome formation, the proteins that have already completed their function must detach from the phagophore. LC3II is one of the few proteins that remains on the phagophore membrane. Adaptor proteins are required to bind the target to be degraded to LC3II. Protein p62 (SQSTM1) is one of the adaptor proteins. It is involved in the regulation of various signaling pathways, since it can bind to polyubiquitinated proteins, which are components of a number of signaling pathways, and induce their degradation in the autophagosome [7].

**Stage V. **Autophagolysosomes are formed as a result of autophagosome and lysosome fusion with the participation of a complex of proteins. HOPS is the main complex. It is composed of the proteins VPS16, VPS41, VPS18, VPS11, VPS39, RILP, and Rab7. This complex is responsible for the fusion of autophagosome and the lysosome membranes [8]. The SNARE/SNAP25 complex, which consists of the proteins Syntaxin, SNAP25 (SNAP27), and Synaptobrevin, is also required for membrane fusion.

**Stage VI. **At this stage, the target is degraded by the lysosomal enzymes inside the autophagolysosome. The same enzymes that performed substrate degradation eventually degrade the autophagolysosome.

**1.1. Role of mTORc1**

The mTORc1 complex, which consists of the proteins mTOR, PRAS40, Deptor, Raptor, and mLST8, is the main regulator of autophagy (*Fig. 1*). Multiple signaling pathways, with PI3K/AKT/mTOR being the main one, regulate mTORc1 activity. Positive regulation of mTORc1 involves an active protein Rheb, which is repressed by the TSC1/2 complex. AKT acts as a negative regulator of TSC1/2 and thus functions as one of the main kinases responsible for autophagy regulation [9].

**1.2. Role of calcium**

Calcium can act as both an inducer and a repressor in autophagy regulation.

The inhibitory effect of Ca^2+^ ions is implemented through their ability to activate calpain, a calcium-dependent cysteine protease that degrades autophagyinitiating proteins (Atg5, Beclin1, and PTEN).

Activation of the PHLPP1β phosphatase due to an increased calpain level leads to the suppression of the ERK1/2 and AKT activities, which disrupts lysosome function. The intracellular protein calpastatin is a natural calpain inhibitor.

**Fig. 2:**
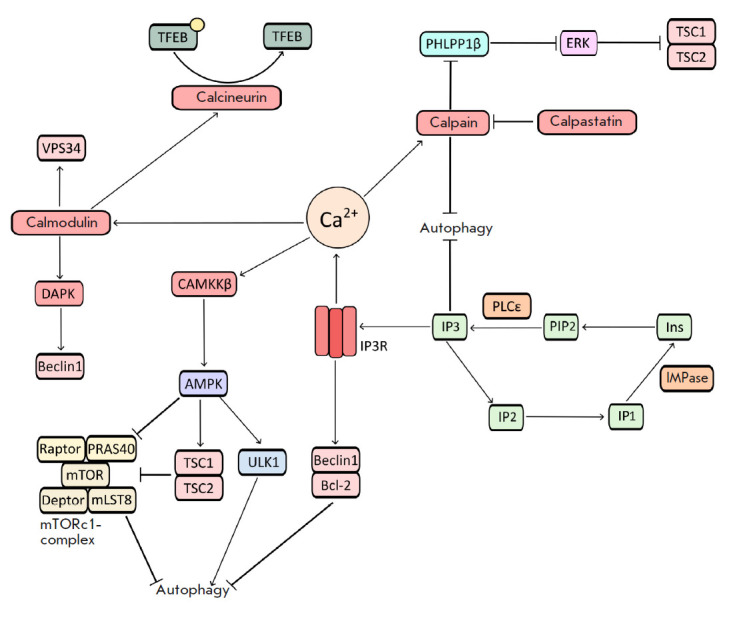
Schematic presentation of the effect of Ca^2+^ ions on autophagy regulation

The action of Ca^2+^ ions on autophagy is implemented through CaMKKβ kinase-mediated activation of AMPK. AMPK inhibits the mTORc1 complex and activates the TSC1/2 and ULK1 proteins. Calmodulindependent protein calcineurin is another calcium target. This protein dephosphorylates the transcription factor TFEB, which results in its activation (*Fig. 2*). Activated TFEB regulates the expression of genes encoding such autophagy proteins as LC3, Beclin1, and p62. Calmodulin activates Vps34 and calmodulindependent kinase DAPK, which is a direct inducer of Beclin1 [10].

The calcium ion level in the cytoplasm is regulated by the activity of calcium channels, including IP3R. IP3R is an ER calcium channel; its function directly depends on the level of IP3, which opens the channel. Channel opening leads to calcium release from the ER into the cytosol. IP3R has an anti-autophagic effect, since it can inhibit the dissociation of the Beclin1/ Bcl-2 complex, thereby reducing the level of active unbound Beclin1 in the cell. The PLC and IMPase proteins are involved in the regulation of the intracellular concentration of IP3; they convert phosphoinositol diphosphate (PI2P) to inositol triphosphate (IP3) and inositol monophosphate (IP1) to inositol, respectively. Inositol can be converted to its original state, PI2P [10].

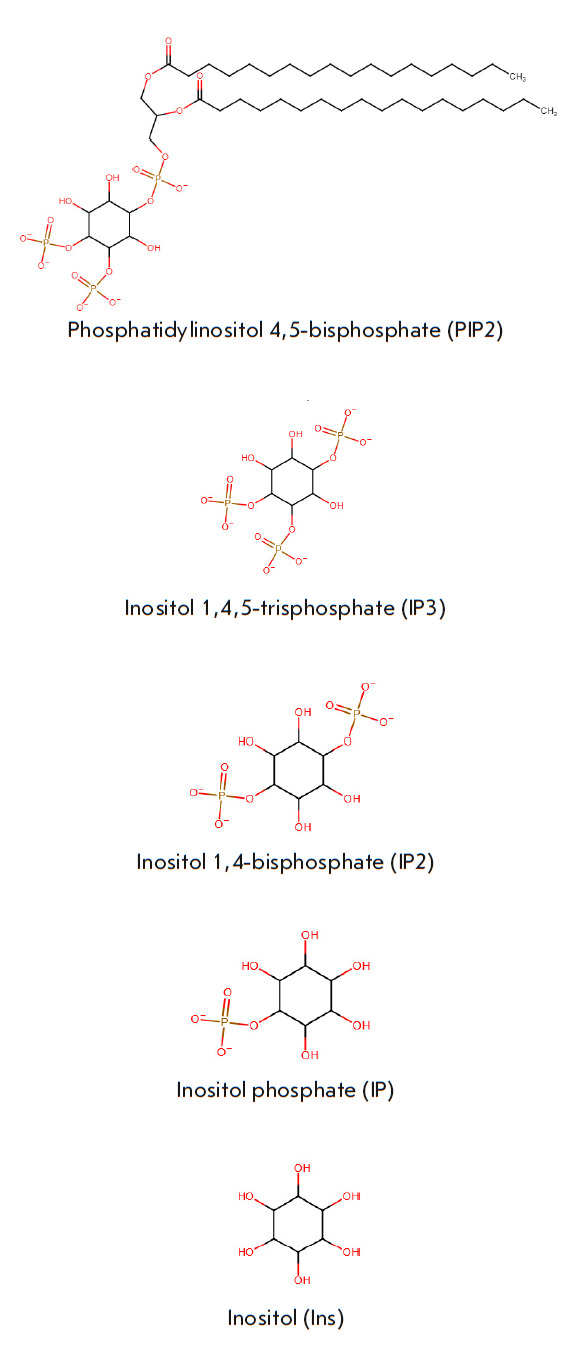



The tumor suppressor p53 plays an important role in autophagy regulation. Depending on the molecular target it interacts with, p53 can act either as an autophagy activator or an inhibitor. The direct interaction of p53 with anti-apoptotic Bcl-2 family proteins (Bcl-2, Bcl-Xl, Mcl-1) inhibits their activity and induces apoptosis. In particular, the interaction of p53 with Bcl-2 causes dissociation of the Bcl-2/Beclin1 complex, with subsequent Beclin1 release and autophagy initiation [11]. An example of p53-mediated autophagy induction is the activation of TSC1/2 and Beclin1 by direct interaction between p53 and DAPK, a Beclin1 activator. The p53 protein can also interfere with autophagy initiation by disrupting ULK complex assembly through binding to the FIP200 protein. The p53 protein can also inhibit AMPK, one of the important autophagy activators (*Fig. 2*) [12].

Autophagy, despite its adaptive function, can be the cause of autophagy-dependent cell death (lethal autophagy), which is characterized by the appearance of a significant number of vacuoles in cells [13].

One of the possible mechanisms of lethal autophagy is the activation of ceramide synthase 1 (CerS1), which forms ceramide on the outer mitochondrial membrane. This causes mitochondrial degradation, since ceramide interacts with the LC3II receptor anchored on the autophagosomal membrane. Excessive accumulation of ceramide on the mitochondrial membrane was found to significantly increase the risk of lethal autophagy induction [13].

Target degradation can also occur without the formation of an autophagosome and other specific vesicles; this pathway is called chaperone-associated autophagy [14]. It begins with the formation of a transmembrane channel by the oligomeric lysosomal protein LAMP2A (CD107). This channel materiliazes upon the occurrence of a so-called misfolded protein, which has an abnormal conformation and contains a unique KFERQ motif, in the cytosol. To form the channel, the KFERQ motif of the misfolded protein must recruit a complex of proteins including HSC70, which acts as a chaperone [10, 14]. The misfolded protein then passes through the LAMP2A channel into the lysosome, where it is degraded.

## 2. ROLE OF AUTOPHAGY IN DISEASE DEVELOPMENT

Autophagy is involved in the development of a number of human diseases (atherosclerosis, diabetes mellitus, ischemia of different localization, hepatocirrhosis, chronic obstructive pulmonary disease, etc.), including both disease onset and response to it.

**2.1. Autophagy and neurodegenerative diseases**

Neurodegenerative diseases form an extensive group of pathologies caused by neuronal cell death. The mechanisms underlying the development of these diseases are not fully clear. However, it is known that they are usually associated with the production and accumulation of agglomerates of misfolded proteins with an abnormal structure both in the intercellular space and in cells. Both central and peripheral nervous system cells can be involved in neurodegeneration, which causes gradual impairment of motor, psychological, and cognitive functions.

**Fig. 3:**
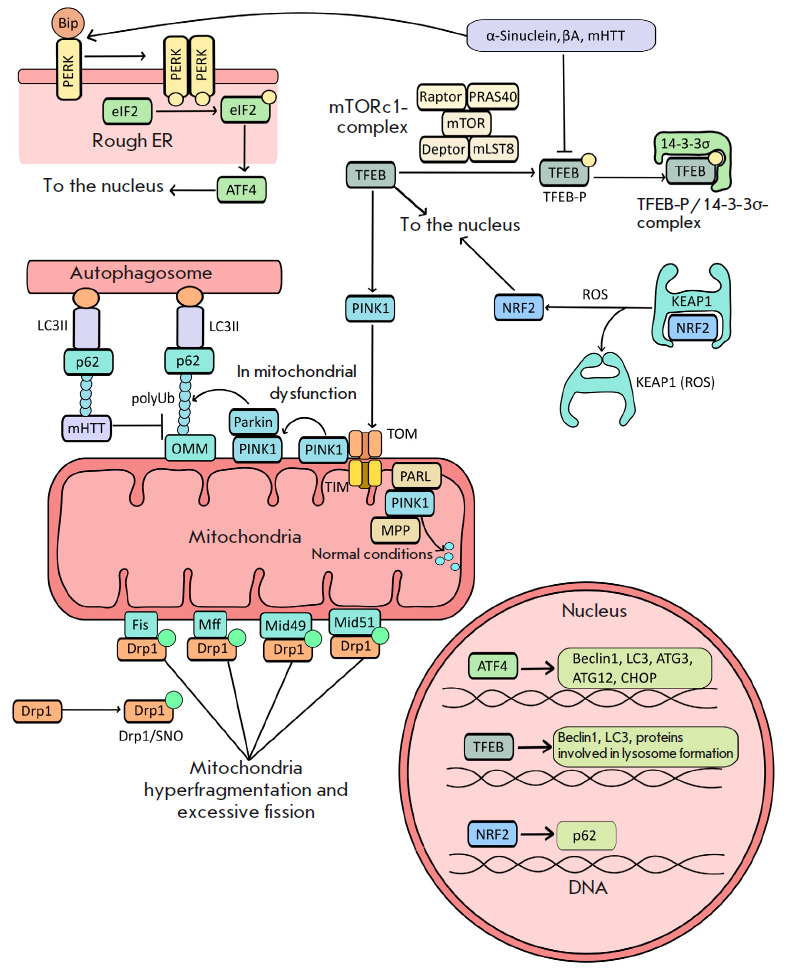
Effect of ER stress-induced misfolded proteins on the regulation of TFEB activity and the mTORc1 complex in neurodegenerative diseases (α-synuclein, β-amyloid, and mHTT in Parkinson, Alzheimer, and Huntington diseases, respectively). The PERK/Bip heterodimeric complex is located on the ER membrane. The complex of chaperone Bip and PERK prevents ER stress activation. The Bip protein recognizes misfolded proteins and delivers them to proteasomes for degradation. Bip binds to misfolded proteins, leading to PERK release followed by its dimerization and activation. The PERK dimer phosphorylates the translation initiation factor eIF2, resulting in inactivation of the latter and inhibition of the translation of many proteins, except for the ATF4 transcription factor, which migrates to the nucleus and activates autophagy gene expression

There are many mechanisms that aim to eliminate misfolded proteins in the cell, including autophagy. Autophagy can be either triggered by ER stress induction, in particular, the PERK/eIF2A/ATF4- signaling pathway, as a response to the production of misfolded proteins (*Fig. 3*) or mediated by chaperones [15]. Autophagy can also participate in the elimination of misfolded proteins characteristic of a specific neurodegenerative disease [1]. Impaired removal of misfolded proteins leads to their accumulation and further aggregation in bodies and plaques. Lewy bodies (α-synuclein) are formed in cells in Parkinson disease, while senile (β-amyloid) and neurofibrillary (tau protein) plaques are produced in Alzheimer disease.

Autophagy impairment in neurodegenerative diseases is accompanied by the accumulation of lysosomes and immature autophagosomes in neurons. This phenomenon is associated with impaired inactivation of the TFEB transcription factor, which regulates the expression of many genes encoding autophagy proteins (LC3, Beclin1, p62, etc.) and the proteins involved in lysosome biogenesis.

In normal conditions, the mTORc1 complex plays a significant role in the regulation of TFEB activity; it inhibits TFEB translocation to the nucleus by phosphorylating it, which results in the formation of the 14-3-3σ/TFEB(P) complex in the cytoplasm. In neurodegenerative diseases, the activity of the mTORc1 complex drops, which results in TFEB release from the 14-3-3σ/TFEB(P) complex and its translocation to the nucleus (*Fig. 3*). Misfolded proteins were found to prevent TFEB inactivation, thus significantly increasing the expression of the genes they regulate. In neurodegenerative diseases, this can be considered a cell compensatory response to a decrease in autophagy efficiency [16].

TFEB is also involved in the regulation of PINK1, a serine/threonine kinase responsible for the localization of ubiquitin ligase Parkin on the mitochondrial outer membrane (MOM) due to a decrease in the membrane potential of damaged mitochondria. Parkin polyubiquitinates MOM proteins, leading to the formation of the OMM/polyUb/p62-LC3II protein complex. This complex is required to recruit autophagosomes to mitochondra for the degradation of the latter in the autophagolysosome. Mitochondrial degradation in the autophagolysosome is called mitophagy. In normal conditions, PINK1 is transported to the mitochondrial matrix by TOM/TIM translocases, where it is degraded by the proteases PARL and MRR (*Fig. 3*) [16].

*2.1.1. Huntington disease. *Huntington disease is an autosomal-dominant disease. Its early stage is characterized by neurodegeneration of basal brain structures (striatum), while disease progression leads to complete atrophy of the cerebral cortex. The first symptoms appear at the age of 35–45 years. At early disease stages, motor functions are impaired, and cognitive and mental abnormalities can also be observed. Mental disorders such as aggression, depression, panic attacks, etc. develop during disease progression. Memory impairment and motor disorders become pronounced; bradykinesia, ataxia, and decreased reflexes are observed. Death occurs 15–20 years after the diagnosis. To date, there are no drugs to treat Huntington disease.

The pathogenesis of Huntington disease is associated with the expansion of CAG trinucleotide repeats in the *HTT *gene, which codes for the huntingtin (Htt) protein. In normal conditions, the number of such repeats does not exceed three. The expansion of repeats has a cumulative nature; i.e., the more there are repeats in *HTT*, the higher the risk of disease development is; however, ≥40 CAG repeats are considered a critical threshold [17, 18]. In normal conditions, Htt participates in the axonal transport and acts as an adaptor protein to kinesin. The mutant Htt protein (mHtt) lacks the ability to recruit kinesin to the vesicle, resulting in impaired vesicular transport through the axon [19]. The mutant protein mHtt can interact with transcription factors such as CREB, CBP, TFIID, p53, and SP1 [19], disrupting their DNA-binding activity. This leads to reduced production of vital proteins. Unlike wild-type Htt, mHtt can induce autophagy gene expression through the ER stress activation (the PERK/eIF2A/ATF4 signaling pathway) (*Fig. 3*) [15]. In addition, mHtt can enhance TFEB dephosphorylation, leading to an increased transport of the latter into the nucleus and expression of genes encoding autophagy proteins (*Fig. 3*).

It was found that mHtt can directly bind to Beclin1 thus impairing the PI3KC3 complex assembly and autophagy initiation [18]. Thus, mHtt can affect autophagy activity through different pathways. Disruption of mHtt degradation due to impaired autophagy leads to its accumulation in the cell cytoplasm with the formation of protein aggregates, which ultimately results in a more aggressive disease course [15, 19].

Accumulation of mHtt in the cytoplasm can result in its association with p62 and disruption of LC3II function on the autophagasome membrane. This affects autophagosome formation around the target to be degraded, resulting in the formation of empty autophagosomes, with a possibility to induce cell necroptosis [19, 20].

*2.1.2. Alzheimer disease and the role of autophagy in its development. *Alzheimer disease (AD) is the most common type of senile dementia. Its early stages are characterized by impaired short-term memory and cognitive decline. As the disease progresses, a loss of communication functions, self-care ability, and speech impairment, up to complete aphasia, are observed.

The mechanisms of AD pathogenesis are not fully understood. There are several hypotheses describing the mechanisms underlying AD, including the tau hypothesis and the hypothesis of the accumulation of senile plaques. The role of mitochondria in AD has also been actively investigated.

The role of β-amyloid in AD pathogenesis has not been established yet; recent studies have questioned the theory of the leading role of senile plaques in neurodegeneration [21]. Beta-amyloid (βA) is a polypeptide consisting of 42 amino acid residues; stacks of these polypeptides form senile plaques. Beta-amyloid is formed by amyloidogenic proteolytic cleavage of the β-amyloid precursor protein (APP), whose gene is located on chromosome 21. APP participates in cell adhesion and contributes to cell survival. APP cleavage resulting in βA formation is mediated by β-secretase (BACE1) and γ-secretase. The C-terminus of APP is cleaved by γ-secretase, while its N-terminus is cleaved by β-secretase. Proteolytic cleavage of APP yields βA monomers, which form extracellular protein conglomerates: so-called senile plaques [22]. These structures spatially interfere with the formation of synaptic connections and initiate local inflammation due to the release of numerous pro-inflammatory factors by microglia cells. This inflammation is caused by the interaction of the transmembrane receptor TREM2 with amyloid plaques, resulting in the activation of the NF-κB and Syk-kinase proteins, which are involved in the activation of cytokines and other inflammatory factors (IL-2 and NO synthases), and neuronal death [23].

Tau protein, which is a member of the microtubule- associated protein (MAP) family, is also involved in AD pathogenesis. MAP proteins provide microtubule rigidity and stiffness. This is achieved through the ability of tau to bind to tubulin and form “stiffening ribs” along microtubules. The efficiency of this binding depends on the tau phosphorylation level. The higher the phosphorylation level of the tau protein is, the lower its affinity for tubulin. In normal conditions, two to three amino acid residues are phosphorylated in the tau protein. Some tau protein mutations increase its phosphorylation level. For example, four missense mutations (G272V, P301L, V337M, and R406W) cause tau overphosphorylation and its detachment from microtubules. Tau detachment results in its accumulation in the cytoplasm, followed by its export into the intercellular space and aggregation in neurofibrillary tangles [24]. The condition characterized by such aggregation is called tauopathy [25].

There has been a growing body of evidence of the involvement of mitochondria in AD. APP accumulates in mitochondria as a result of its transfer from the cytoplasm to the mitochondrial intermembrane space by translocase TOMM40, where APP inhibits the cytochrome oxidase complex (complex IV of the electron transport chain), which reduces ATP production [26]. Beta-amyloid also binds to cyclophilin D, which is involved in the regulation of calcium levels in mitochondria and mitochondrial gene expression. As a consequence, disruption of cyclophilin D function decreases mitochondrial gene transcription and impairs the mitochondrial functioning [27].

Beta-amyloid is known to affect mitochondrial fission. During mitochondrial fission, Drp1 proteins form a ring-like structure around the organelle. Accumulation of βA triggers the production of inducible NO synthase (iNOS) in the cell, which is involved in the S-nitrosylation of Drp1 (Drp1/SNO) (*Fig. 3*). This modification disrupts the regulation of Drp1 oligomerization on the mitochondrial wall, resulting in abnormal fragmentation and increased mitochondrial number [28, 29]. Drp1 is recruited to the mitochondrial membrane through association with the adaptor transmembrane proteins Fis1, Mff, and Mid49/51 (*Fig. 3*) [29].

Autophagy is involved in APP degradation in vesicles. Autophagy impairment in AD is associated with the accumulation of a large number of immature autophagosomes in neurons due to a dysfunction of the ESCRT-III complex (cytosolic protein complex) involved in the formation of multivesicular bodies (MVBs). This complex is responsible for the transport of ubiquitinated membrane proteins to MVB. Inhibition of MVB formation makes their fusion with the autophagosome impossible. It also abrogates the formation of the late endosome and further destruction of APP in lysosomes [30, 31]. The direct effect of autophagy on AD pathogenesis is associated with Atg7, which is involved in βA transport to MVB. Atg7 participates in the accumulation of amyloid agglomerates in exosome vesicles and transportation of these agglomerates to the intercellular space. It was experimentally shown that suppression of the Atg7 activity by small interfering RNAs decreases βA production in neurons. Atg7 deficiency was shown to lead to a significant accumulation of hyperphosphorylated tau. Thus, Atg7 is actively involved in tau protein degradation and, therefore, can be directly involved in its turnover [31]. The evidence suggests that the proteins associated with autophagy regulation can be involved in AD development.

Another AD trait is the accumulation of reactive oxygen species (ROS) in neurons. ROS production is mediated by NADPH-oxidase 4 (NOX4), which is activated by the interaction of the transmembrane protein RAGE with βA molecules. It is important to note that ROS can both activate and inhibit autophagy.

An example of a positive effect of ROS on autophagy is the activation of the ROS-KEAP1-NRF2-p62 signaling pathway. ROS oxidize cysteine residues in KEAP1, which forms the heterodimeric KEAP1/NRF2 complex. Oxidation of KEAP1 residues leads to the release of the NRF2 transcription factor, which enhances the expression of the p62-encoding gene (*Fig. 3*) [32].

The negative effect of ROS on autophagy is associated with a decrease in HIF-1α activity. In the active state, this transcription factor enhances the transcription of the LC3, BNIP3/NIX, and REDD genes by interacting with their enhancers. Upon ROS accumulation in cells, the proline residues in HIF-1α are oxidized, resulting in HIF-1α polyubiquitination and its further proteolysis [33].

**2.2. Autophagy and autoimmune diseases**

There are numerous causes behind autoimmune diseases. Their development is associated, on the one hand, with the formation of a pool of mature B cells (plasmocytes) producing autoreactive antibodies, and, on the other, with a decrease in either the activity or number of regulatory T cells [34]. The occurrence of a pool of autoreactive lymphocytes can be associated with a disrupted selection of the entire pool of lymphocytes in the major immune organs. The subsequent defense response to autoreactive lymphocytes involves their elimination through interaction with the epithelial cells of the medullary region of the thymus stroma. These cells produce tissue-specific antibodies that interact with autoreactive lymphocytes, which ultimately leads to their death. This process is called autoresistance. Impaired autoresistance in autoimmune diseases is believed to maintain the pool of autoreactive lymphocytes.

In addition to maintaining a pool of autoreactive lymphocytes that are abnormally aggressive towards normal cells, the pathological immune response can be associated with impaired degradation of damaged or dead cell fragments, components of pathogenic microorganisms, and other antigens, followed by their accumulation due to impaired autophagy [35].

Autophagy also promotes the assembly of the MHC complexes involved in antigen presentation on the cell membrane. These complexes act as immune response activation signals. Autophagy disruption leads to impaired MHC II assembly, since no pathogen fragmentation in the autophagolysosome or interaction of the resulting fragments with MHC II takes place [35].

There are also mechanisms in which autophagy acts as a negative regulator of autoimmune processes. In particular, autophagy affects immune cell survival and differentiation. This is evidenced by the fact that Atg5 dysfunction in B lymphocytes leads to impaired differentiation of pro-B cells into pre-B cells. B cells with mutant inactive Atg5 are less viable than cells with wild-type Atg5. Autophagy can also affect the BCR signaling pathway required for B cell activation. Apoptotic B cells with an activated BCR signaling pathway are characterized by an abnormal increase in autophagosome formation, which leads to cell death. This evidence indicates that autophagy can be involved in the inhibition of autoreactive B cells [36, 37].

Autophagy is one of the processes mediating T cell viability. Inhibited formation of components of the autophagy initiator complex PI3KC3-C1 in T cells is known to result in impaired removal of damaged organelles, impaired differentiation, and all-out death [38]. In addition, T cell survival is reduced in Atg7, Atg5, and Atg3 deficiency.

*2.2.1. Crohn disease and the role of autophagy in its pathogenesis. *Similar to many autoimmune diseases, the pathogenesis of Crohn disease, which is manifested by chronic inflammation of the large intestine, has not been fully elucidated. The intestinal mucosa in Crohn disease resembles a cobblestone sidewalk and has characteristic thickened areas. Symptoms are similar to dyspeptic disorders; they include abdominal pain, diarrhea, anorexia, nausea, vomiting, and weight loss. The inflammation area can spread to the entire gastrointestinal tract, up to the oral mucosa. The absorption of nutrients in the intestine is impaired in disease. The molecular mechanism of Crohn disease has not been revealed; therefore, there are no effective ways to treat it [39].

To date, there are several hypotheses on the mechanism of Crohn disease onset and progression. According to one of them, mutations in the gene encoding the NOD2 receptor play a key role.

**Fig. 4:**
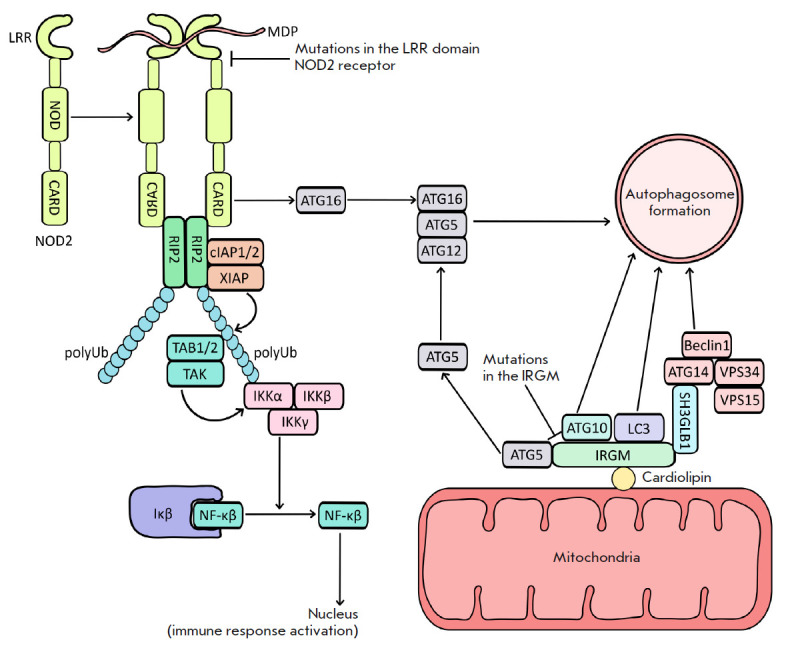
Crohn disease and autophagy. Schematic presentation of intracellular receptor NOD2 activation and signaling pathways affecting autophagosome assembly and interaction with mitochondria (details can be found in the article)

NOD2 is a cytosolic receptor protein located on the inner side of the cytoplasmic membrane. It is involved in the antibacterial immune response. NOD2 contains three distinct domains: NOD, LRR, and CARD. Muramyl dipeptide (MDP), a component of bacterial cell wall peptidoglycan, is a NOD2-activaing ligand. Receptor activation leads to simultaneous interaction of MDP with the LRR domains of two NOD2 molecules, resulting in their dimerization (*Fig. 4*). This causes NOD2 activation and recruitment of two RIP2 molecules to the CARD domain. An E3 ubiquitin ligase complex containing cIAP1/2 and XIAP associates with RIP2, leading to complex activation and polyubiquitin formation on RIP2. A complex consisting of the TAB1/2 and TAK proteins is formed on polyubiquitin, initiating the assembly of the IKKα/β/γ complex, which participates in the phosphorylation of Iκβ, which, in turn, forms a complex with NF-κβ. This leads to NF-κβ release and activation, followed by its migration to the nucleus [40]. NOD2 also regulates the activity of α- and β-defensins, which form “holes” on the bacterial membrane, eventually leading to cell death.

Mutations in the LRR domain have been found to impair the immune response and increase the chances of survival of intracellular pathogenic bacteria. It ultimately results in increased production of the cytokine IL-23, leading to enhanced chemotaxis of Th17 cells to the intestinal mucosa [41, 42].

Association of Atg16L with NOD2 yields the Agt12/ Atg5/Atg16L complex, which is necessary for the formation of an autophagosome surrounding the bacterium and further bacterial lysis. This subtype of autophagy is called xenophagy. If the LRR domain of NOD2 carries a mutation, Atg16L is not recruited to the membrane. This leads to impaired autophagosome formation and promotes the survival of pathogenic bacteria inside the cell [41, 42].

Another protein involved in Crohn disease pathogenesis is the IRGM (immunity-related GTPase family M) protein, which possesses GTPase activity. This protein binds to the MOM by interacting with the cardiolipin on its surface. IFN-γ synthesis in the cell, as well as cell infection with Gram-negative bacteria, enhances IRGM activity. IRGM is involved in the regulation of antibacterial immune mechanisms in the cell. Active IRGM initiates autophagy through its interaction with the following autophagosome assembly proteins: Atg5, Atg10, Bif-1, LC3, SH3GLB1, UVRAG, Beclin1, and Vps34 (*Fig. 4*) [43].

Inactivating *IRGM *mutations are known to increase the risk of Crohn disease. Introduction of a deletion in the *IRGM *promoter region and an increase in the amount of microRNA-196 targeting IRGM mRNA were shown to reduce autophagy activity [44].

**2.3. Autophagy and cancer**

Similar to other diseases, autophagy has a dual effect on cancer development. On the one hand, autophagy serves as one of the sources of nutrients for rapidly dividing cancer cells. On the other hand, it can inhibit cell division and even cause cancer cell death [45].

Hypoxia develops in the tumor due to a lack of adequate blood supply resulting from aggressive, uncontrolled growth of cancer cells. The metabolism of cancer cells is altered; glycolysis and subsequent anaerobic catabolism are activated. HIF-1α plays a key role in cell adaptation to tissue hypoxia; the HIF-1α transcription factor enhances angiogenesis in tumor, triggers glycolysis, and activates cellular adaptation processes. Disruption of oxygen-dependent proteolysis of HIF-1α in cancer cells impedes its degradation, which results in its accumulation in the cytosol. This, in turn, leads to increased expression of the genes encoding autophagy proteins (Beclin1 and BNIP3) [33, 45].

Depletion of energy reserves in cancer cells leads to the activation of the AMP kinase AMPK, which is induced by ATP deficiency. AMPK is a sensor of the lack of cell energy resources. Activated AMPK phosphorylates Beclin1 and ULK1 at S93, S96, and T388 and at S467, S555, T574, and S637, respectively, leading to their activation. AMPK is also involved in the phosphorylation of mTORc1 complex proteins, causing complex inactivation (*Fig. 2*). These processes are an adaptation to nutrient deficiency; they lead to an increase in autophagy activity and nutrient acquisition by eliminating cancer cell components [46].

The adaptor protein p62 is involved in the autophagosome- mediated degradation of the toxic substances formed during metabolism in cancer cells (*Fig. 1*). A decrease in the p62 level impedes cancer growth. A high p62 level is detected in pancreatic, lung, and liver cancer cells [47].

In addition to the positive effects of autophagy on cancer cell survival, there are also examples of its negative effect. For instance, autophagy can inhibit cancer cell growth and cause cancer cell death through the interaction of Beclin1 with a mutant EGFR tyrosine kinase which is involved in carcinogenesis. The Beclin1–mEGFR interaction inhibits the mitotic activity of the mutant receptor, resulting in suppressed cancer cell growth. In addition, introduction of an inactivating mutation in *BECN1*, which codes for Beclin1, and experimental reduction of its expression lead to enhanced cancer cell growth [48]. A monoallelic deletion in *BECN1 *is often found in breast, prostate, and ovarian cancers. This mutation is found in 40–75% of all the above pathologies and is most common in breast cancer. Beclin1 protein deficiency has also been observed in kidney cancer, non-small cell lung cancer, and cholangiocarcinoma. Hyperexpression of the gene encoding Bcl-2, which can form a complex with Beclin1 (Beclin1/Bcl-2) inhibiting autophagy, has been observed in various lymphomas [49].

*2.3.1. Follicular lymphoma. *Lymphoma is a lymphatic system disease; its development is caused by uncontrolled growth of lymphocytes in the major immune organs and lymph nodes. One variant of follicular lymphoma is non-Hodgkin lymphoma [50]. This disease is characterized by slow progression. Symptoms are observed at late disease stages and include enlargement of lymph nodes in the groin region, neck, and armpits, as well as back pain and intoxication. Displacement of immunocompetent cells and development of immunodeficiency take place during disease progression.

The pathogenesis of follicular lymphoma is associated with the t(14;18)(q32;q21) chromosomal translocation, which is characterized by a rearrangement between the chromosome 18 region encoding the antiapoptotic protein Bcl-2 and the chromosome 14 region encoding the enhancer region of the immunoglobulin heavy chain. This translocation results in a fusion gene expressing Bcl-2 at an abnormally high rate. As noted above, Bcl-2 accumulation leads to excessive recruitment of Bcl-2 to Beclin1, BNIP3, and other autophagy- associated proteins. This results in autophagy inhibition in cells carrying the mutation [49]. One of the main characteristics of the Bcl-2 protein is its anti-apoptotic activity. Accumulation of this protein leads to a decrease in the apoptosis of transformed immature B cells and an enlargement of their pool. Another mutation, which is often detected in lymphomas, is associated with the *Bcl-6 *gene: t(3;14)(q27;q32). In this mutation, a rearrangement of fragments between chromosomes 3 and 14 takes place, which results in a gene sequence encoding mutant Bcl-6 that fails to function properly; i.e., it performs normal differentiation of B cells [51].

In addition, the p62 and LC3II levels are decreased in follicular lymphoma cells, resulting in autophagy inhibition and autophagy-associated cell death [52].

## CONCLUSIONS

Autophagy plays an important role in cells. Autophagy impairment is associated with the development of various diseases, while autophagy activity can affect the course of various diseases in different ways. It should be noted that, despite the active study of the role of autophagy in various cell processes and diseases, the contribution of the individual signaling pathways related to autophagy remains poorly understood and is of great interest.
